# Spatial and Seasonal CH_4_ Flux in the Littoral Zone of Miyun Reservoir near Beijing: The Effects of Water Level and Its Fluctuation

**DOI:** 10.1371/journal.pone.0094275

**Published:** 2014-04-07

**Authors:** Meng Yang, Xuemeng Geng, John Grace, Cai Lu, Yi Zhu, Yan Zhou, Guangchun Lei

**Affiliations:** 1 School of Nature Conservation, Beijing Forestry University, Beijing, China; 2 School of Geosciences, The University of Edinburgh, Edinburgh, United Kingdom; DOE Pacific Northwest National Laboratory, United States of America

## Abstract

Wetlands, and especially their littoral zones, are considered to be CH_4_ emissions hotspots. The recent creation of reservoirs has caused a rapid increase in the area of the world’s littoral zones. To investigate the effects of water depth and water level fluctuation on CH_4_ fluxes, and how these are coupled with vegetation and nutrients, we used static closed chamber and gas chromatography techniques to measure CH_4_ fluxes in the littoral zone of a large reservoir near Beijing, China, from November 2011 to October 2012. We found that CH_4_ flux decreased significantly along a transect from open water to dry land, from 3.1 mg m^−2^ h^−1^ at the deep water site to approximately 1.3 mg m^−2^ h^−1^ at the shallow water site, and less than 0.01 mg m^−2^ h^−1^ in the non-flooded area. Water level influenced CH_4_ flux by affecting soil properties including soil redox potential, soil carbon and nitrogen, and bulk density. The largest emission of all was from the seasonally flooded site after a flooding event (up to 21.1 mg m^−2^ h^−1^), which may have been caused by vegetation decomposition. Submerged sites had greater emissions, while the driest site had lower emissions. Immediately after the monthly measurements had been made, we removed the aboveground vegetation to enable an assessment of the gas transportation per unit of biomass. Removal of biomass decreased emissions by up to 53%. These results indicated the dominant effect of water depth on CH_4_ flux through effects of soil conditions, plant species composition and distribution. This study suggests that temporally flooded wetlands, including littoral zones, contribute significantly to the global CH_4_ burden. However, the current challenge is to capture their spatial extent and temporal variation in the fluxes.

## Introduction

Methane is the second most important contributor to total greenhouse gas emissions, with a Global Warming Potential of 25 over a 100-year time-span [1]. Wetlands are considered to be the most important CH_4_ source, thought to account for 24–39% of the total global emission of CH_4_, albeit with a large degree of uncertainty [2]. Reservoirs are an important type of wetland, whose combined area across the globe has increased in recent years to now occupy approximately 5×10^5^ km^2^, which is 1/3 the size of all natural lakes [3]. One reason for the large development of reservoirs is their potential as a clean energy source, although uncertainties in their methane emissions have led some scientists to question whether they are as clean as people believe [4]. Several authors have insisted that the subject of greenhouse gas emissions from reservoirs requires more scientific study [5]–[7]. The Ministry of Water Resources of the People's Republic of China has stated that China has over 80,000 reservoirs, large and small [8]. However, work in China on their greenhouse gas emissions, and the extent to which these reservoirs contribute to total emissions, is limited [9].

The littoral zone of reservoirs features fast and complicated material cycles and is reported to be a hotspot for CH_4_ emissions [10], [11]. Furthermore, there are indications from elsewhere about the importance of the littoral zone: for example, in boreal systems, where about 70% of total CH_4_ emissions comes from the littoral zone, even though its area is no more than 24% of the total wetland area [12]. Water depth and its diurnal and seasonal fluctuations are thought to be the main drivers determining the characteristics of the littoral habitat, including plant species composition and distribution, soil conditions, and CH_4_ flux [11], [13]–[16]. However, the interaction between water level fluctuation, CH_4_ production, transportation, and emissions is still not fully understood [17], [18], especially the effect of dynamic water fluctuation coupling with vegetation amd nutrients [19], [20]. The rate of change of the water table, as distinguished from just differences in the water table, is known to be a major environmental factor controlling the CH_4_ flux [21].

In summary, studies of the CH_4_ flux from the littoral zone are essential for evaluating the atmospheric and climatic impacts of reservoirs as energy sources, as well as for a better understanding of the biogeochemical mechanism of CH_4_ flux. Research on the mechanisms of how the water level and its fluctuations influence CH_4_ flux is an important foundation for related work aimed at modeling and controlling carbon loss through ecosystem management. In this context, we report the seasonal and monthly variation of CH_4_ flux of the littoral zone of Miyun Reservoir, Beijing, China. We hypothesized that water depth and water level fluctuation would be the main factors driving the CH_4_ flux variability, together with soil and vegetation.

## Materials and Methods

This study was authorized by Beijing North Miyun Reservoir Eco-agriculture Co. Ltd. It did not harm any protected species, and thus no ethics committee was required to authorize the work. All data used in this paper were collected in the field by the research team, and the raw data are available upon request from the senior author. No data were downloaded from publicly available resources, and thus no permission was required.

### Study area

The research was carried out at Miyun Reservoir (40°29′N, 116°50′E), which is located in the northern mountainous area near Beijing, China. It was built in 1960 with a maximum water area of 188 km^2^. Its catchment is characterized by warm temperate semi-humid monsoonal climate with an annual average air temperature of 10.5°C, maximum air temperature of 38°C, and a minimum of −18°C. The reservoir is normally covered by ice from the middle of November to the end of March. The growing season is from April to November. The annual average precipitation is close to 600 mm, of which 80% is concentrated from July to August [22]. The annual change in the water level is 1–5 m because of rainfall and water supply for domestic use. The area between the highest and lowest water level from 1984 to 2005 was 84 km^2^ [23]. In the summer of 2012, when the work was carried out, continuous heavy rain in July caused a sudden water level increase of one meter, and part of the littoral vegetation was inundated.

**Figure 1:**
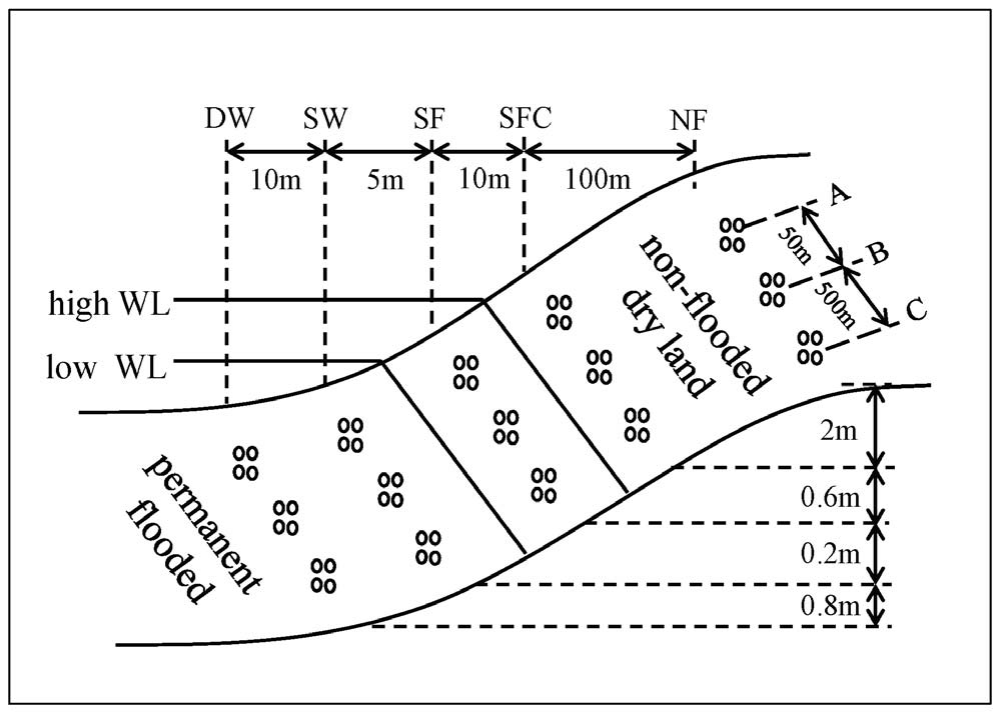
Experimental design. WL: water level. The sites are grouped at different heights. DW: deep water site; SW: shallow water site; SF: seasonally flooded site; SFC: ‘control site’ for the seasonally flooded site; NF: non-flooded site. A, B and C denote samples from different vegetation types within each height band (see Table 1 for information on plant species). There were four replicates in each case, repeatedly sampled six times (also repeatedly sampled seven times in a day) in the year. For more details on water depth and other environmental parameters, see Figures 2 and 3.

**Figure 2:**
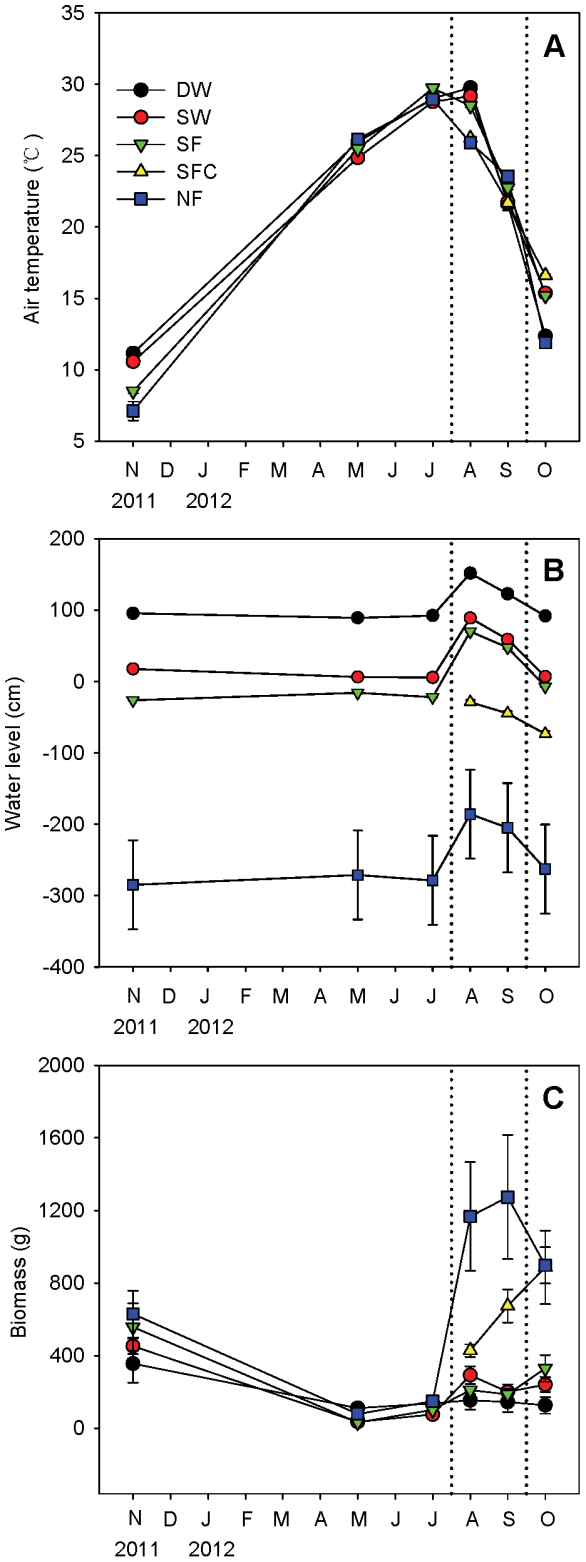
Monthly variation of air temperature, water level and biomass of each site. Days between dotted lines was the high water level period and thus the seasonally flooded site (SF) was under water. DW: deep water site; SW: shallow water site; SF: seasonally flooded site; SFC: ‘control site’ for the seasonally flooded site; NF: non-flooded site. A negative value of water level indicates the ground water depth. Error bars represent SE.

**Figure 3:**
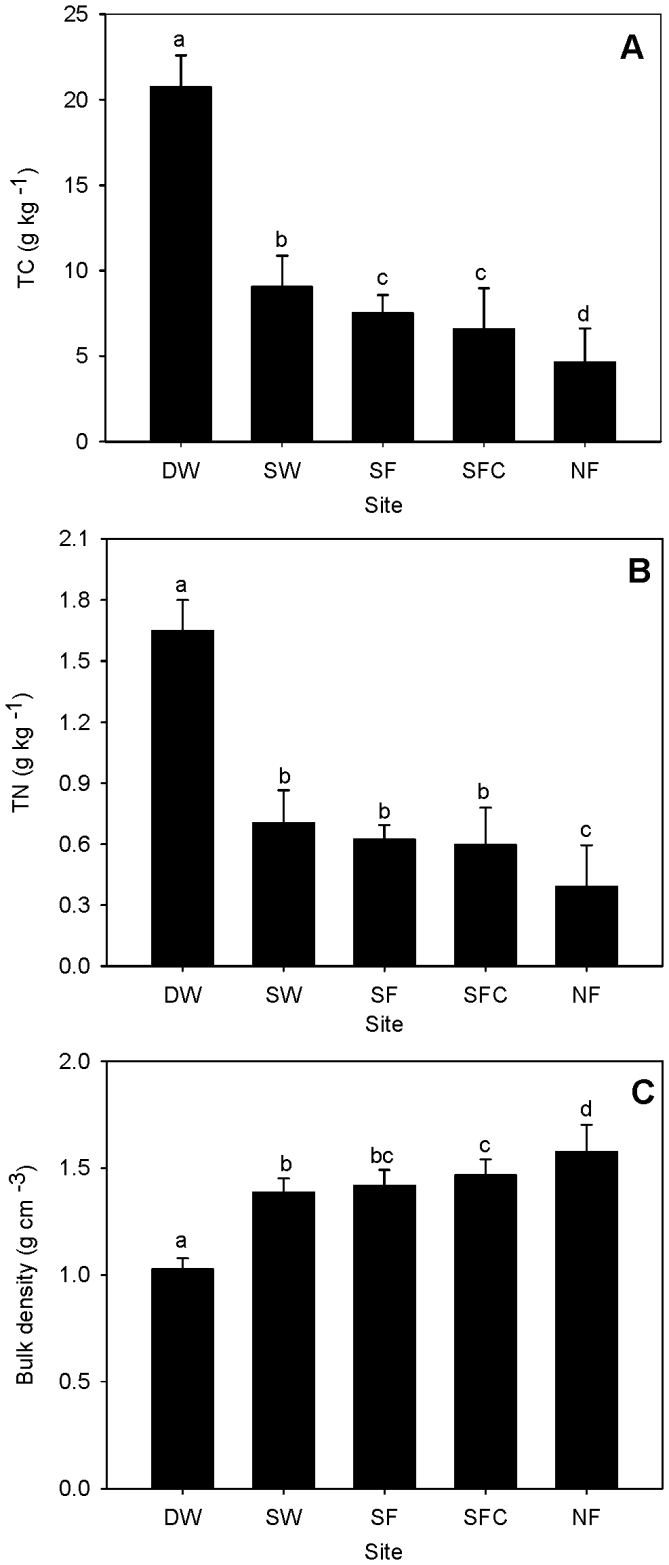
Soil properties (mean ± SE) of each site. Bars with different letters indicate a significant difference at p<0.05. TC: total carbon, TN: total nitrogen. DW: deep water site; SW: shallow water site; SF: seasonally flooded site; SFC: ‘control site’ for the seasonally flooded site; NF: non-flooded site.

We divided the littoral zone into five areas based on water level (Figure 1). Sites were selected ranging from locations in open water to the dry area on higher ground, to provide five contrasting environments: (i) deep water area (DW); (ii) shallow water area (SW); (iii) seasonal (August and September) flooded area (SF); (iv) ‘seasonally flooded control’ (SFC) area, which was 500 m away from SF, had the same plant species and similar soil carbon/nitrogen content as SF, but escaped the flood in August and September because of its 1-m-higher elevation; and (v) permanent non-flooded area (NF). Details of the water depths in each of these areas are shown in Figure 2B. Three typical plant communities in each area were selected, and Table 1 shows the dominant species in different seasons in all zones. For more details on biomass and soil, see Figures 2 and 3.

**Table 1:** Dominant plant species at each plot in different months.

Site		Nov 2011	May 2012	Jul 2012	Aug 2012	Sep 2012	Oct 2012
DW	A	*Echinochloa olonum* ^AE^	*Myriophyllum* sp.		*Trapa* ^AE^ sp.		
	B	no vegetation					
	C	*Typha angustifolia* ^AE^					
SW	A	*Xanthium sibiricum* ^E^	*Scirpus planiculmis* ^AE^		*Echinochloa colonum* ^AE^		
	B	*Setaria viridis* ^E^	*Bidens pilosa* ^E^		*Echinochloa colonum* ^AE^		
	C	*Zea mays* ^E^	*Polygonum lapathifolium* ^E^				*Typha angustifolia* ^AE^
SF	A	*Xanthium sibiricum*	*Cirsium setosum*		*Cirsium setosum* ^E^		*Cirsium setosum*
	B	*Setaria viridis*	*Hemarthria altissima*		*Hemarthria altissima* ^E^		*Hemarthria altissima*
	C	*Zea mays*	*Polygonum lapathifolium*		*Polygonum lapathifolium* ^E^		*Polygonum lapathifolium*
SFC	A	#			*Cirsium setosum*		
	B	#			*Hemarthria altissima*		
	C	#			*Zea mays*		
NF	A	*Xanthium sibiricum*					
	B	*Setaria viridis*	*Artemisia argyi*				
	C	*Zea mays*					

# indicates no data;

DW: deep water site, SW: shallow water site, SF: seasonally flooded site, SFC: ‘control site’ for seasonally flooded site, NF: non-flooded site;

A, B, C indicates sample plot with different vegetation;

Species with aerenchyma are denoted ^A^, species that are emergent are denoted ^E^.

### CH_4_ flux measurements

Methane flux was measured in November 2011, then May, July, August, September and October 2012. The experiment of three plots at site SFC was carried out just after the flooding and during the time when the water level dropped from August to October 2012. In order to reduce uncertainty in the average daily flux, sampling to capture any diurnal variation was performed at three-hourly intervals (local time: 6, 9, 12, 15, 18, 21 and 24 h). Each plot had four replicates located within three meters from each other. To eliminate disturbance to the soil, wooden access platforms were built.

The static opaque chamber technique was used to determine the CH_4_ flux [24]. The chambers were made of stainless steel (volume: 125 L; surface area: 0.25 m^2^) and covered with polyethylene foam to avoid any warming effect inside the chamber. An internal chamber (volume: 200 L; surface area: 0.25 m^2^) could be added if plants were tall. Two fans were built into the chamber for air mixing. Four gas samples (200 mL each) were taken using 100-mL polypropylene syringes at 15-min intervals over a 45-min period after enclosure, and stored in 500-mL plastic and aluminum membrane gas sampling bags. The concentration of CH_4_ was analyzed within one week by gas chromatography (7890A, Agilent, California, USA) equipped with a flame ionization detector (FID). Gases were separated with a column (3 m, 3.2 mm) packed with Porpak Q (80/100 mesh). The temperatures of the oven, injector, and detector were 70°C, 20°C, and 200°C, respectively. The flow rate of the carrier gas (N_2_) was 25 mL min^−1^. Standard CH_4_ gas (2.03 ppm in air, China National Research Center for Certified Reference Materials, China) was used for precision verification for CH_4_ concentrations. The coefficient of variation was below 1%. The flux of CH_4_ was calculated following Chen et al. [25]. In order to determine the effect of aboveground vegetation on flux, one more flux measurement was taken at 9 am the following day (after seven times sampling for diurnal variation), with aboveground plant material removed. Chambers were reset into new positions near the old positions each sampling month. All positions at each site were within an area of 20 m^2^, but not so close to each other to cause artifacts in the data through (for example) changes in the local hydrology.

### Environmental factors

In order to analyze the effects of environmental parameters on fluxes, the following factors were taken into account: water level, dissolved oxygen (DO) in water, soil total carbon (TC) and nitrogen (TN), soil bulk density, biomass, and air temperature.

Water level was measured after gas sampling at DW, SW and SF (when SF had standing water in August and September 2012). At site SF (when there was no standing water in November 2011, May, July and October 2012) and SFC, a 1-m PVC tube was inserted vertically into the soil under the chamber after all monthly gas sampling was complete, allowing two hours for the water level to equilibrate before measuring the level. The water table of site NF was calculated according to the elevation measured by a Global Navigation Satellite System receiver (BLH-L90, Daheng International, China). DO in water was measured during the gas sampling by a handheld multi-parameter meter (Professional Plus, YSI, USA), after flooding.

Soil samples at site DW, SW, SF and NF were collected from three different layers (0–10 cm, 10–20 cm, and 20–30 cm below ground) at each replicate location in November 2011, except site SFC in October 2012. After air-drying and grinding (passing through a 100 mesh sieve), TC and TN were analyzed using an elemental analyzer (vario MACRO cube, Elementar, Germany). Soil bulk density was measured following Chinese national standards NY/T 1121.4-2006 [26].

To determine which species had aerenchyma tissues, thin hand sections were made of roots and stems (photomicrographs were made). The aboveground biomass of every replicate in the chamber was weighed after drying at 80°C to constant mass.

Diurnal air temperature was measured by a digital thermometer (JM624, Jinming, China) at the start and end of each gas sampling at every plot.

### Statistical analysis

Reported daily fluxes were obtained by averaging the means of the three-hourly values. To estimate how much CH_4_ was transported by vegetation and to calculate the differences between species, plant-mediated flux was estimated on the basis of the difference between the flux before and after vegetation removal. We followed Kankaala et al. [27] to derive a mass-based plant transport index, PTI:
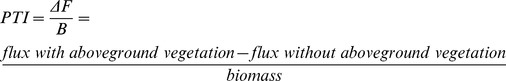



The Kruskal–Wallis H test was used to test for spatial difference of flux and the Mann–Whitney U test for further multiple comparisons. Spatial differences of soil properties were analyzed with one-way ANOVA, and then LSD for multiple comparisons. A log_10_ transformation was used to show the correlation between water depth and CH_4_ flux. A value of 0.5 was added to the flux data before transformation to make sure that all of the data were positive. Spearman analyses were used for correlations between flux and environmental factors. All the analyses above were performed using IBM SPSS Statistics (19.0, IBM, USA). Charts were made using SigmaPlot (11.0, SYSTAT, USA).

## Results

### Spatial and seasonal variation of CH_4_ flux

**Figure 4:**
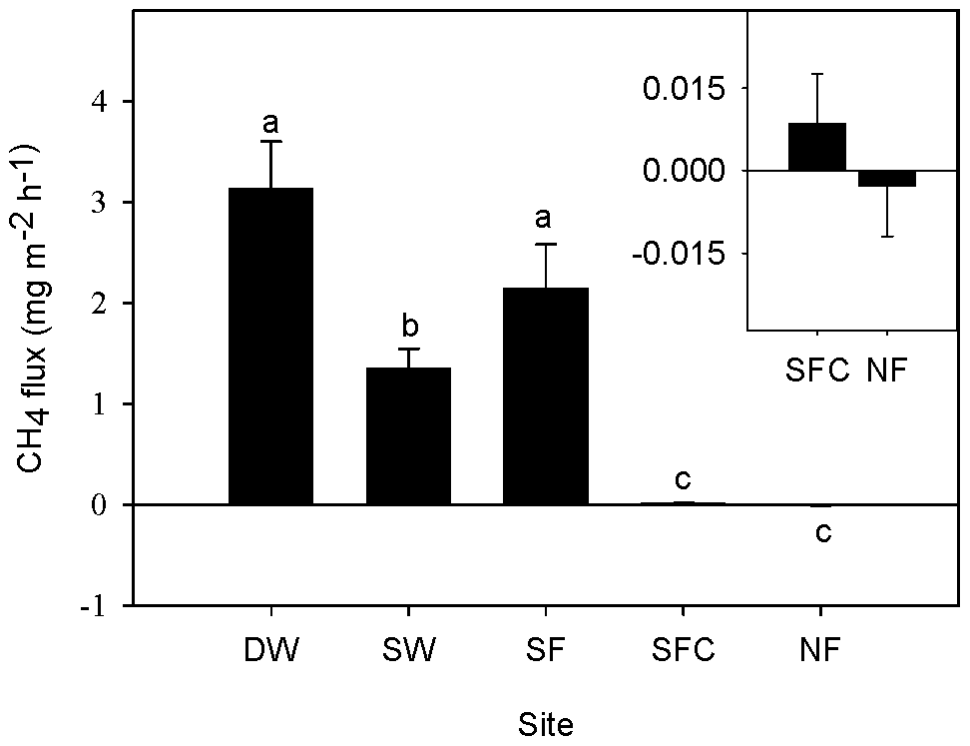
CH_4_ flux (mean ± SE) at different sites. Bars with different letters indicate a significant difference at p<0.05. Positive values of flux indicate a CH_4_ source. Inset in the top right-hand corner shows SFC and NF on a different scale. DW: deep water site; SW: shallow water site; SF: seasonally flooded site; SFC: ‘control site’ for the seasonally flooded site; NF: non-flooded site.

CH_4_ fluxes at the five sites showed significant differences (n = 324, p<0.05; Figure 4). CH_4_ fluxes at permanently flooded sites, both the deep and shallow water sites (DW: 3.1±0.5 mg m^−2^ h^−1^; SW: 1.3±0.2 mg m^−2^ h^−1^), and the seasonally flooded site (2.1±0.4 mg m^−2^ h^−1^) were significantly higher than non-flooded sites. The seasonally flooded site SF, which was flooded for two months, emitted higher (1.6 times) CH_4_ than the permanent flooded site SW with shallow water. The seasonally flooded site SF was dramatically higher than its control site, SFC (the flux was 247 times higher). Although the never-flooded site NF presented as a weak sink (−2.7±9.2 10^−3^ mg m^−2^ h^−1^), while site SFC was a weak source (8.7±8.9 10^−3^ mg m^−2^ h^−1^), there was no significant difference between them (n = 144, p>0.05). The average CH_4_ emission from all the flooded sites (DW, SW and SF) was about 1120 times higher than the non-flooded sites (SFC and NF).

**Figure 5:**
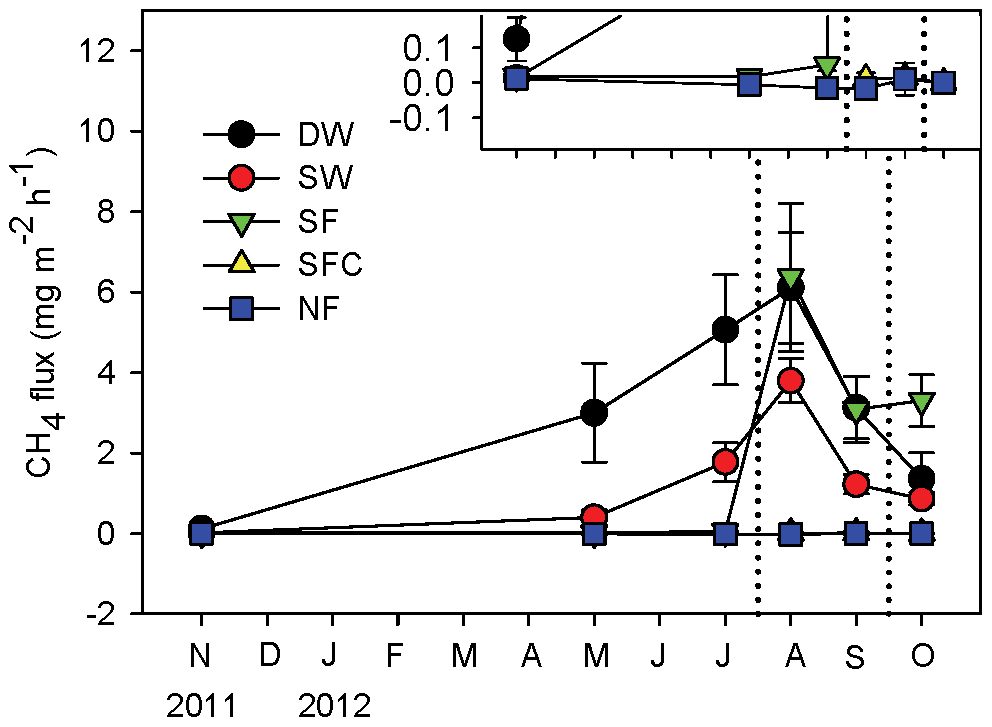
Monthly variation of CH_4_ flux (mean ± SE) at different sites. A positive value of flux indicates a CH_4_ source. Inset in the top right-hand corner shows SFC and NF on a different scale. Days between dotted lines was the high water level period and thus the seasonally flooded site (SF) was under water. DW: deep water site; SW: shallow water site; SF: seasonally flooded site; SFC: ‘control site’ for the seasonally flooded site; NF: non-flooded site.

There were different seasonal patterns among different sites (Figure 5). CH_4_ flux at site DW and SW continuously increased from November 2011 to August 2012, and thereafter decreased gradually. CH_4_ flux at site SF increased slightly from November 2011 to July 2012. After flooding it rose sharply (from 0.05 mg m^−2^ h^−1^ to 6.4 mg m^−2^ h^−1^) to a high peak, and then remained the highest emitter among all sites until the autumn. The seasonal pattern of site NF was totally contrary to sites DW, SW and SF; although the amplitude of flux was narrow. It decreased from autumn to the following summer, and then increased gently. CH_4_ flux at site SFC was as low as site SF before it was flooded.

### Effects of environmental factors

**Figure 6:**
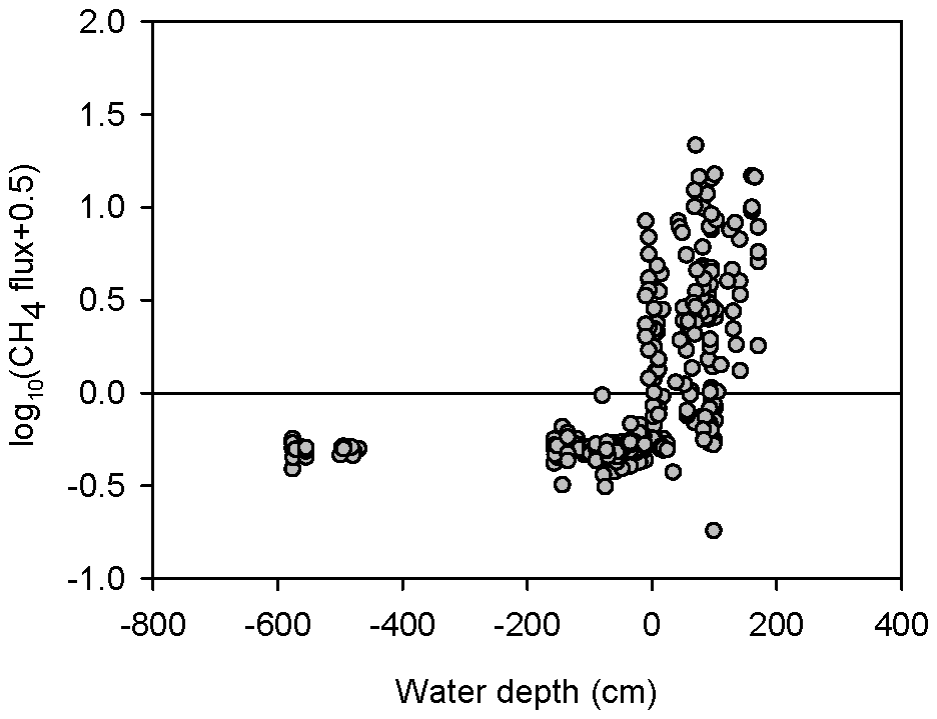
Relationship between water depth and CH_4_ flux.

Considering all the cases where the water depth was 2 m or less, the water depth was positively correlated with CH_4_ flux (n = 324, p<0.01; Table 2). When there was standing water (water depth >0 cm; Figure 6), the habitat always emitted more CH_4_ than when there was no standing water (water depth <0 cm).

**Table 2:** Correlation coefficient *R* between CH_4_ flux and environmental parameters.

Site	Water level	Soil TC	Soil TN	Soil bulk density	Biomass	Water DO	Air temperature
all sites	0.75**(324)	0.62**(324)	0.56**(324)	−0.53**(324)	−0.26**(324)	−0.28**(168)	0.25**(324)
DW	0.38**(72)	0.45**(72)	0.43**(72)	−0.02(72)	0.42**(72)	−0.31**(72)	0.63**(72)
SW	0.42**(72)	−0.06(72)	−0.05(72)	−0.03(72)	0.08(72)	−0.32**(72)	0.65**(72)
SF	0.74**(72)	0.19(72)	0.12(72)	0.07(72)	0.24*(72)	−0.51*(24)	0.08(72)
SFC	0.47**(36)	0.57**(36)	0.57**(36)	0.24(36)	−0.31(36)	#	0.10(36)
NF	−0.02(72)	−0.14(72)	−0.15(72)	0.05(72)	−0.03(72)	#	−0.28*(72)

# indicates no data;

Numbers in () indicate the sample size;

TC: total carbon, TN: total nitrogen, DO: dissolved oxygen;

** indicates significant correlation (*P* <0.01), * indicates significant correlation (*P*<0.05).

Soil properties varied according to the zone: TC and TN were highest at site DW and lowest at site NF, while bulk density showed an opposite trend (n = 60, p<0.05; Figure 3). Soil TC and TN were positively correlated with flux at all sites (n = 324, p<0.01; Table 2), as well as individually at sites DW and SFC (n = 72, p<0.01). Bulk density was negatively correlated with flux at the whole-site scale (n = 324, p<0.01; Table 2), and no significant correlations were found at separate sites (n = 72, p>0.05).

**Figure 7:**
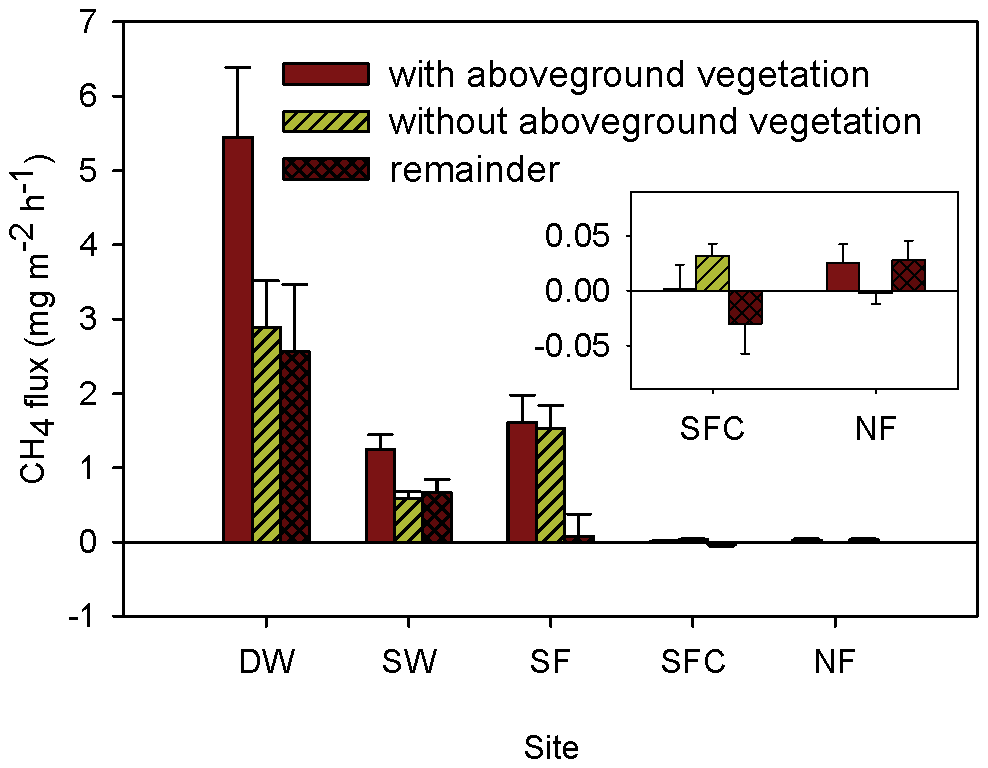
Contribution of aboveground plants to CH_4_ flux (mean ± SE) of each site. A positive value of flux indicate a CH_4_ source. A positive remainder means methane is transported from the vegetation to the atmosphere. Inset in the right-hand center shows SFC and NF on a different scale. DW: deep water site; SW: shallow water site; SF: seasonally flooded site; SFC: ‘control site’ for the seasonally flooded site; NF: non-flooded site.

**Figure 8:**
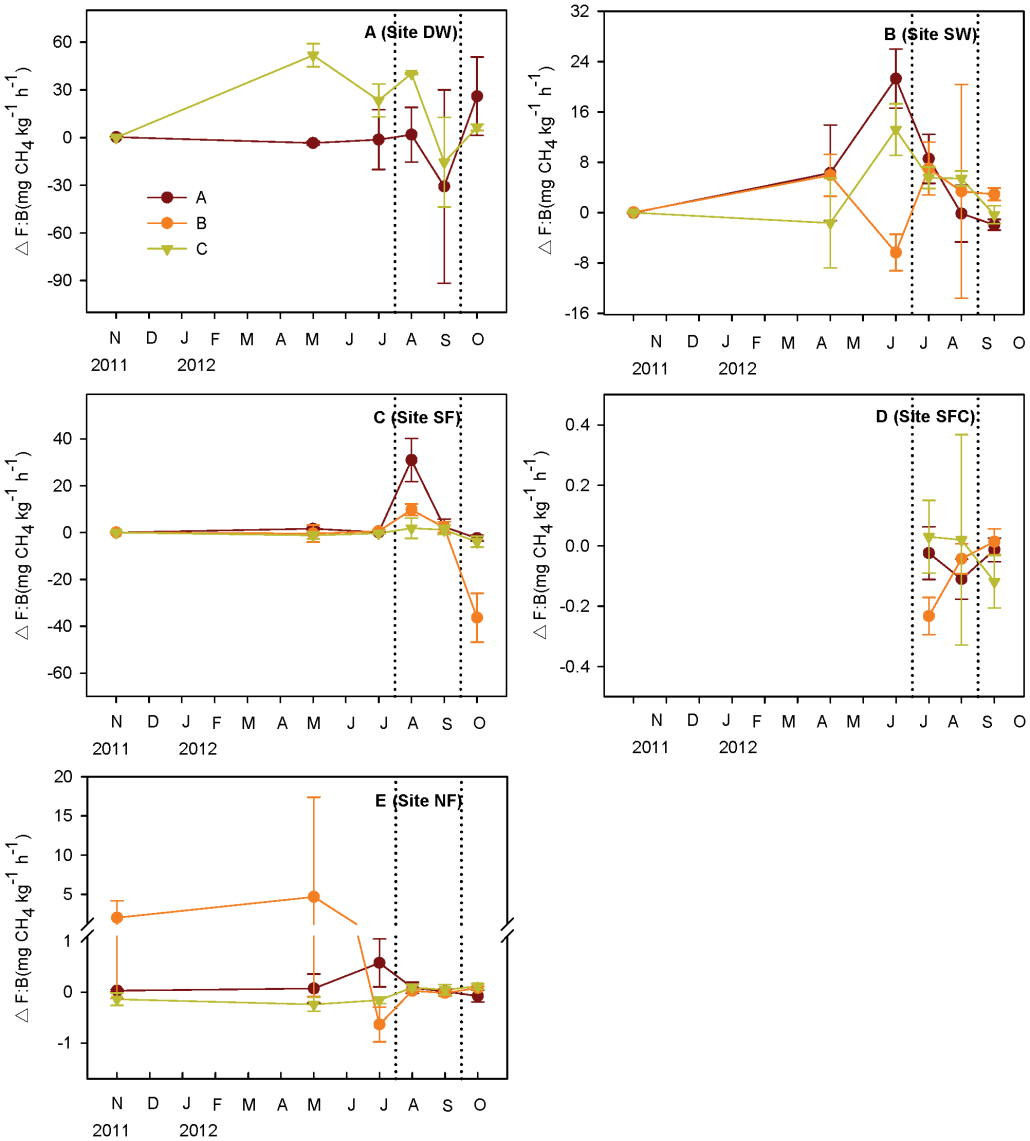
Above-ground plant transport index (mean ± SE). △F (mg CH_4_ m^−2^ h^−1^) is the flux with vegetation minus the flux without vegetation, i.e. the flux of methane that can be attributed to the presence of the vegetation. B (kg m^−2^) is biomass. A positive flux means methane is transported from the vegetation to the atmosphere. Days between dotted lines was the high water level period and thus the seasonally flooded site (SF) was under water. DW: deep water site; SW: shallow water site; SF: seasonally flooded site; SFC: ‘control site’ for the seasonally flooded site; NF: non-flooded site.

There were large differences in CH_4_ flux before and after the removal of aboveground vegetation at all sites (Figure 7). After vegetation removal, fluxes at sites DW and SW decreased by approximately 50% (47% and 53%, respectively), while the contribution of transportation at site SF was much smaller (6%). For the two emergent sites, SFC and NF, where fluxes were very small, opposite patterns were found. After the plants were removed, emissions at site SFC increased, while at site NF they decreased. The plant transportation index differed among the five sites and 14 plots (Figure 8). It showed seasonal variation, with the highest transportation efficiency found in spring or summer at most of the sites.

**Figure 9:**
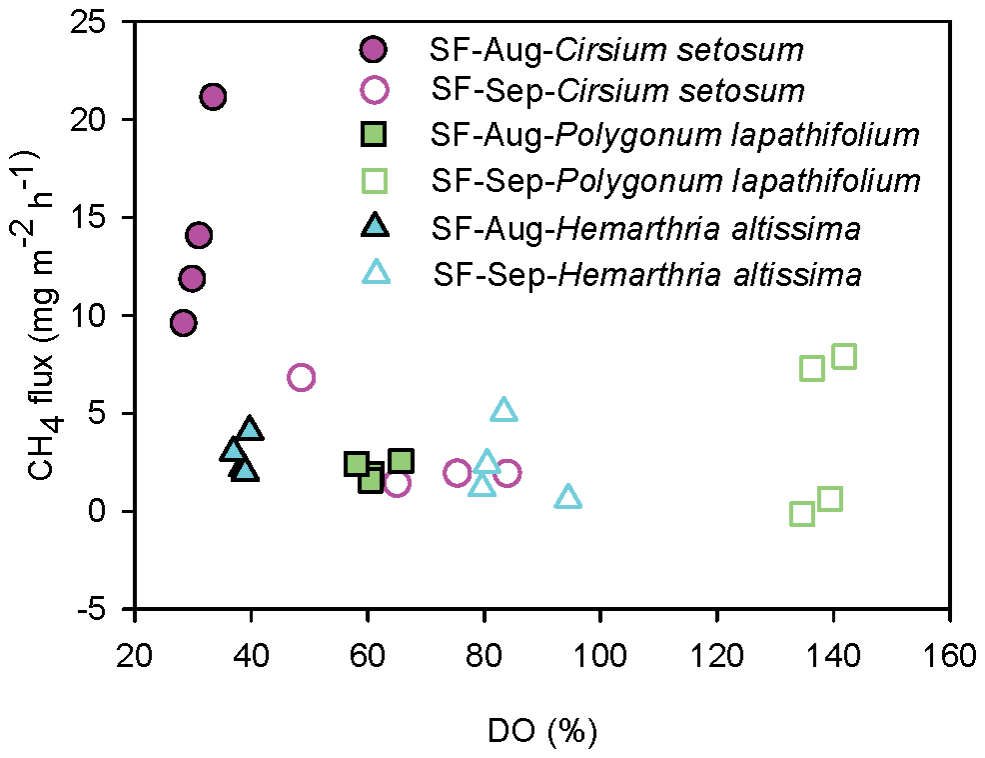
Relationship between dissolved oxygen (DO) of water and flux of the seasonally flooded site (SF).

CH_4_ emissions showed different patterns with different plant species after flooding at site SF (Figure 9). High CH_4_ emissions (from 9.6 mg m^−2^ h^−1^ to 21.1 mg m^−2^ h^−1^; average: 14.2±2.5 mg m^−2^ h^−1^) were recorded at the plot with *Cirsium setosum* after the flooding (August; flooded for 20 days), which was 6.8 and 5.1 times higher than the site with *Polygonum lapathifolium* (from 1.6 mg m^−2^ h^−1^ to 2.6 mg m^−2^ h^−1^; average: 2.1±0.2 mg m^−2^ h^−1^) and *Hemarthria altissima* (from 2 mg m^−2^ h^−1^ to 4.1 mg m^−2^ h^−1^; average: 2.8±0.5 mg m^−2^ h^−1^), respectively. At the same time, the lowest water DO (30%–33%) was observed at the plot with *C. setosum* when the flux was the highest. Water DO was negatively correlated with flux at all flooded sites (DW: n = 72, p<0.01; SW: n = 72, p<0.01; SF: n = 24, p<0.01; Table 2).

The vegetation biomass began to increase in July and peaked in September to October (Figure 2C). There was a significantly negative correlation (n = 324, p<0.01; Table 2) between flux and biomass when all sites were taken as a whole. However significantly positive correlations were observed at sites DW and SF (n = 72, p<0.01; n = 72, p<0.05). No significant correlations were found at the other three sites (n = 72, p>0.05).

Air temperature positively correlated with CH_4_ flux at sites DW and SW (n = 72, p<0.01; Table 2), but was negatively correlated at site NF (n = 72, p<0.05). No significant correlations were observed at sites SF and SFC (n = 72, p>0.05; n = 36, p>0.05).

## Discussion

### High CH_4_ emissions from the littoral zone

The average CH_4_ emission rate (1.3 mg m^−2^ h^−1^) from the littoral zone of Miyun Reservoir was 6.5 times higher than the flux from the open water area (0.2 mg m^−2^ h^−1^) [28]. The low emissions from open water are similar to those from reservoirs at higher or lower latitudes, but in those cases the littoral zone has not been investigated [29], [30]. Reservoirs differ enormously in the percentage of their area that can be deemed ‘littoral’. In the case of Miyun, in the period from 1984 to 2005, the average area of the water surface was 103±15 km^2^, while its annul difference was 9.5±2 km^2^ [23]. Although the seasonally flooded area is as high as 8.5% of the total area of the reservoir, it explains how the littoral component of emissions is as much as 40% of the total. The high level of emissions from the littoral zone emphasizes its status as a methane hotspot in this case, but not all reservoirs will have a littoral zone comprising such a high percentage of the total. Although the present study involving 15 typical habitats, there are still uncertainties in the estimation because of high spatiotemporal flux variation and environmental heterogeneity, as demonstrated by the complex effects of plant, soil and temperature on fluxes found in this research. Furthermore, the dynamics of the littoral zone exacerbates the uncertainty. Nevertheless, emissions from the littoral zone are likely to form a substantial part of the reservoir’s methane budget, and deserve further research [10], [31].

### Water level fluctuation and increased CH_4_ emissions from the littoral zone

After 20 days of flooding, CH_4_ emissions from site SF increased sharply to the highest level among all sites, and this high rate lasted to October (Figure 5). In contrast, a decreasing of CH_4_ emissions was reported at a site in Finland and was explained by diminished plant-mediated transportation and decreased carbon supply from growing plants to the rhizosphere after plants were totally submerged [16]. In another study, different patterns were shown when flooding occurred in different seasons; CH_4_ emissions were negligible after flooding in autumn or winter, but increased sharply after flooding in summer [32]. Based on the present work, we suggest that anaerobic plant decomposition, which apparently occurred at site SF after flooding, is the main reason for high emissions. Elsewhere, it has been shown to be the cause of CH_4_ loss from tropical reservoirs [33]. Our situation was similar, but one in which the effect was more rapid due to the herbaceous environment.

Different fluxes among flood-tolerant and -intolerant plants were observed. In the case of the flood-intolerant *C. setosum*, flooding caused a sharp increase in emissions, but for the flood-tolerant *P. lapathifolium* and *H. altissima*, under the same water depth, no sharp increase was seen. This phenomenon might be the reason for the difference between the aforementioned research in Finland and that of our own; their research was carried out at places where *Carex* is the main vegetation, which does not rot immediately after flooding [16]. Different responses of plant species to flooding help to explain the spatial variation, and this could represent a potential approach to reducing methane emissions, i.e., through careful vegetation management.

The lowest water DO found in the plots with flood-intolerant species (Figure 9) may be the result of oxygen consumption during plant tissue decomposition [34]. Variations in CH_4_ emissions have sometimes been attributed to variations in methane oxidation [35]. The observed negative relationship between DO and CH_4_ flux (Table 2) asserts the importance of available DO and plant decomposition on CH_4_ loss. A dramatic rise in CH_4_ emissions appeared when the DO fell to approximately 30%, suggesting that 30% might be the lower bound of usable DO concentration for methanotrophs. However, more work on this aspect is needed, since CH_4_ oxidation is a complex process affected by many factors, including microorganisms, temperature, water content, and redox potential [36], [37]. It is therefore probable that the DO threshold will vary across different habitat types.

It is conceivable that wetlands featuring a littoral zone constituting a relatively large proportion of the total area, as well as frequent water level fluctuations, might produce especially high emissions when the majority of the plant community comprises flood-intolerant species. It is further conceivable that climatic variability, i.e., an increased risk of flood and drought [38], will exacerbate methane production because of CH_4_ emissions caused by unusual water level fluctuations, and those associated with re-flooding. For example, these conditions occurred in China after an extreme drought in 2010–2011, which caused the drying out of numerous lakes, giving them the appearance of grasslands, exemplified by a well-documented case at the largest freshwater lake in China, Poyang, which has an area of 3,150 km^2^ [39].

### Effects of water depth on the flux through soil and plants

Water depth determines soil and plant conditions, which in turn influence CH_4_ flux through different mechanisms. However, we found these effects to be sometimes limited by the water depth itself.

Flooding decreases the soil redox potential [40]–[42], which affects both methanogenesis activity and gas transfer through plant tissues [43]. Besides, the anaerobic conditions inhibit aerobic respiration and thus allow more organic matter accumulation [44], [45], which was the likely reason for the soil nutrients pattern found in our research (Figure 3). Carbon and nitrogen compounds form the substrate for CH_4_ production [46], [47]. Positive correlations are usually observed between soil nutrients and CH_4_ emissions [48], [49]. However, the insignificant correlations at SW, SF and NF suggested that the effect of soil nutrients was sometimes weak, and even dominated by other factors. A similar result has also been shown at a grassland site in Germany, where flux was found to be positively correlated with soil moisture but not nitrogen fertilizer application from 0 to 450 kg N ha^−1^ yr^−1^ [50]. High accumulation rates of organic matter forms a soil that has a low bulk density [51] and high porosity [52], which might accelerate CH_4_ diffusion in soil pores, leading to methane release [53]. Our results agree with the literature, showing that the water level influenced CH_4_ production through the soil redox potential and availability of organic substrates.

Plants are another important reason for the spatial pattern of flux. An apparent interspecific difference in the plant-mediated flux was found at site DW (Figure 8A), i.e., the flux transported by emergent plants was much higher than that of totally submerged plants. Higher emissions from emergent plants have also been observed at a Qinghai–Tibetan Plateau wetland, although they did not reduce the flux through the water surface [54]. Emergent macrophytes with a well-developed vascular system and with aerenchyma can be an important pathway for CH_4_ transportation from sediment to atmosphere [55]. Although submerged plants have also developed aerenchyma, even a thin film of water over the leaves and stems might inhibit gas release from plant tissues since the diffusive velocity of gases in water is much slower than in air, by a factor of 10^4^ [56]. In such cases, emissions will be suppressed until the time when ebullition begins. Further interpretation in this regard came from a study in which it was found that CH_4_ emissions in a sedge-dominated zone decreased significantly, when the flood level was high enough to submerge the venting structures of the plants [16].

Much variation in plant-mediated CH_4_ emissions among the five sites was observed (Figure 7), decreasing by 99% along the water depth gradient from water to dry land. Around 50% of the CH_4_ emissions was transported by plant tissues at the permanently flooded sites (DW and SW), as was also found in a marsh with *Spartina alterniflora* [57]. Plant-mediated fluxes from the same species growing under different water depths were different, e.g., sites SF and SFC (Figures 9C and 9D). This shows that, although the transportation ability was different among plant species, it was nevertheless highly dependent on flooding.

Besides the spatial pattern, plant development is also considered as an explanation for the seasonal variation of CH_4_ flux. There were positive correlations between flux and the biomass of sites DW and SF (Table 2). Gas transportation through aerenchyma is the most likely explanation [55], [57]. Besides, we also calculated methane flux per kilogram of biomass as an index of transportation efficiency (Figure 8). The arched patterns of plant transportation at sites DW and SW suggest that vegetation activity might be correlated with plant transportation capacity [58]–[60]; and the high level of emissions in summer were caused not only by the high biomass, but were also influenced by the high transpiration rate of vegetation (methane dissolved in plant water can be transported from soil to the leaves, and hence reach the atmosphere). The interaction between biomass and transportation efficiency might be a reason for the lack of correlation between biomass and emissions at sites SW, SFC and NF (Table 2). Taking all sites as a whole, a negative correlation between flux and biomass was observed (Table 2), but this relationship is affected by the fact that the lowest CH_4_ emissions and highest biomass were found at the driest site.

## References

[1] Solomon S, Qin D, Manning M, Chen Z, Marquis M, et al.. (2007) Climate Change 2007: The Physical ScienceBasis. Contribution of Working Group I to the Fourth Assessment Report of the IntergovernmentalPanel on Climate Change. Cambridge University Press, Cambridge, United Kingdom and New York, NYUSA.

[2] Denman KL, Brasseur G, Chidthaisong A, Ciais P, Cox PM, et al.. (2007) Couplings Between Changes in the Climate System and Biogeochemistry. In: Climate Change 2007: The Physical Science Basis. Contribution of Working Group I to the Fourth Assessment Report of the Intergovernmental Panel on Climate Change [Solomon, S., D. Qin, M. Manning, Z. Chen, M. Marquis, K.B. Averyt, M.Tignor and H.L. Miller (eds.)]. Cambridge University Press, Cambridge, United Kingdom and New York, NYUSA.

[3] Wildi W (2010) Environmental hazards of dams and reservoirs. Near Curriculum in Natural Environmental Science 88: 187–197.

[4] Gunkel G (2009) Hydropower - A Green Energy? Tropical Reservoirs and Greenhouse Gas Emissions. Clean-Soil Air Water 37: 726–734.

[5] Mäkinen K, Khan S (2010) Policy considerations for greenhouse gas emissions from freshwater reservoirs. Water Alternatives 3: 91–105.

[6] Li S, Lu X (2012) Uncertainties of carbon emission from hydroelectric reservoirs. Natural Hazards 62: 1343–1345.

[7] Bridgham SD, Cadillo-Quiroz H, Keller JK, Zhuang QL (2013) Methane emissions from wetlands: biogeochemical, microbial, and modeling perspectives from local to global scales. Global Change Biology 19: 1325–1346. 23505021 10.1111/gcb.12131

[8] Chen X (2009) Gazette of the ministry of water resources of the People's Republic of China. Beijing: Ministry of Water Resources of the People's Republic of China.

[9] Hu Y, Cheng H (2013) The urgency of assessing the greenhouse gas budgets of hydroelectric reservoirs in China. Nature Climate Change 3: 708–712.

[10] Chen H, b NW, Yao S, Gao Y, Zhu D, et al (2009) High methane emissions from a littoral zone on the Qinghai-Tibetan Plateau. Atmospheric Environment 43: 4995–5000.

[11] Bergström I, Mäkelä S, Kankaala P, Kortelainen P (2007) Methane efflux from littoral vegetation stands of southern boreal lakes: An upscaled regional estimate. Atmospheric Environment 41: 339–351.

[12] Juutinen S (2004) Methane fluxes and their environmental controls in the littoral zone of boreal lakes. Joensuu, Finland: University of Joensuu.

[13] Light HM, Darst MR, MacLaughlin MT, Sprecher SW (1993) Hydrology, vegetation, and soils of four North Florida river flood plains with an evaluation of state and federal wetland determinations. Tallahassee, Florida: US Department of the Interior, US Geological Survey.

[14] Laanbroek HJ (2010) Methane emission from natural wetlands: interplay between emergent macrophytes and soil microbial processes. A mini-review. Annals of Botany 105: 141–153. 19689973 10.1093/aob/mcp201 PMC2794055

[15] Huang Y, Jiao Y, Zong L, Zheng X, Sass RL, et al (2002) Quantitative dependence of methane emission on soil properties. Nutrient Cycling in Agroecosystems 64: 157–167.

[16] Juutinen S, Alm J, Larmola T, Huttunen JT, Morero M, et al (2003) Methane (CH4) release from littoral wetlands of Boreal lakes during an extended flooding period. Global Change Biology 9: 413–424.

[17] Petrescu A, Van Beek L, Van Huissteden J, Prigent C, Sachs T, et al.. (2010) Modeling regional to global CH4 emissions of boreal and arctic wetlands. Global Biogeochemical Cycles 24, GB4009, DOI: 10.1029/2009GB003610.

[18] Petrescu AMR, van Huissteden J, Jackowicz-Korczynski M, Yurova A, Christensen TR, et al (2008) Modelling CH4 emissions from arctic wetlands: effects of hydrological parameterization. Biogeosciences 5: 111–121.

[19] Gorres CM, Conrad R, Petersen SO (2013) Effect of soil properties and hydrology on Archaeal community composition in three temperate grasslands on peat. FEMS Microbiology Ecology 85: 227–240. 23521431 10.1111/1574-6941.12115

[20] Mander Ü, Maddison M, Soosaar K, Karabelnik K (2011) The Impact of Pulsing Hydrology and Fluctuating Water Table on Greenhouse Gas Emissions from Constructed Wetlands. Wetlands 31: 1023–1032.

[21] Yamamoto A, Hirota M, Suzuki S, Oe Y, Zhang P, et al (2009) Effects of tidal fluctuations on CO2 and CH4 fluxes in the littoral zone of a brackish-water lake. Limnology 10: 229–237.

[22] Gao ZQ (1989) Beijing Chronicles of Water Conservancy,Volume III Beijing: Editing Committee of the Beijing Chronicles of Water Conservancy.

[23] Cao RL, Li CJ, Liu LY, Wang JH, Yan GJ (2008) Extracting Miyun reservoirs water area and monitoring its change based on a revised normalized different water index. Science o f Surveying and Mapping 33: 158–160.

[24] Moore TR, Roulet NT (1991) A comparison of dynamic and static chambers for methane emission measurements from subarctic fens. Atmosphere-Ocean 29: 102–109.

[25] Chen H, Wu N, Wang Y, Zhu D, Yang G, et al (2013) Inter-Annual Variations of Methane Emission from an Open Fen on the Qinghai-Tibetan Plateau: A Three-Year Study. PloS ONE 8: e53878. 23342029 10.1371/journal.pone.0053878 PMC3544678

[26] MAPRC Ministry of agriculture of People’s Republic of China (2007) Available: http://cx.spsp.gov.cn/index.aspx.Accessed 2013 Nov 10.

[27] Kankaala P, Käki T, Mäkelä S, Ojala A, Pajunen H, et al (2005) Methane efflux in relation to plant biomass and sediment characteristics in stands of three common emergent macrophytes in boreal mesoeutrophic lakes. Global Change Biology 11: 145–153.

[28] Yang M, Li HL, Lei T, Zhou Y, Lu C (2011) Spatial-temporal variation of CH_4_ flux and its environmental factors at Miyun Reservoir. Wetland Science 9: 191–197.

[29] Demarty M, Bastien J, Tremblay A (2011) Annual follow-up of gross diffusive carbon dioxide and methane emissions from a boreal reservoir and two nearby lakes in Quebec, Canada. Biogeosciences 8: 41–53.

[30] Zheng H, Zhao X, Zhao T, Chen F, Xu W, et al (2010) Spatial–temporal variations of methane emissions from the Ertan hydroelectric reservoir in southwest China. Hydrological Processes 25: 1391–1396.

[31] Bergier I, Novo EM, Ramos FM, Mazzi EA, Rasera MF (2011) Carbon dioxide and methane fluxes in the littoral zone of a tropical Savanna Reservoir (Corumba, Brazil). Oecologia Australis 15: 666–681.

[32] Boon PI, Mitchell A, Lee K (1997) Effects of wetting and drying on methane emissions from ephemeral floodplain wetlands in south-eastern Australia. Hydrobiologia 357: 73–87.

[33] Abril G, Parize M, Pérez MAP, Filizola N (2013) Wood decomposition in Amazonian hydropower reservoirs: An additional source of greenhouse gases. Journal of South American Earth Sciences 44: 104–107.

[34] Cunha-Santino MBd, Pacobahyba LD, Bianchini I Jr (2010) Decomposition of aquatic macrophytes from Cantá stream (Roraima, Brazil): kinetics approach. Acta Limnologica Brasiliensia 22: 237–246.

[35] Le Mer J, Roger P (2001) Production, oxidation, emission and consumption of methane by soils: A review. European Journal of Soil Biology 37: 25–50.

[36] Chowdhury TR, Dick RP (2013) Ecology of aerobic methanotrophs in controlling methane fluxes from wetlands. Applied Soil Ecology 65: 8–22.

[37] Knittel K, Boetius A (2009) Anaerobic oxidation of methane: progress with an unknown process. Annual Review of Microbiology 63: 311–334. 10.1146/annurev.micro.61.080706.093130 19575572

[38] Kundzewicz ZW, Mata LJ, Arnell NW, Döll P, Kabat P, et al.. (2007) Freshwater resources and their management. Climate Change 2007: Impacts, Adaptation and Vulnerability. Contribution of Working Group II to the Fourth Assessment Report of the Intergovernmental Panel on Climate Change, M.L. Parry, O.F. Canziani, J.P. Palutikof, P.J. van der Linden and C.E. Hanson, Eds., Cambridge University Press, Cambridge, UK.

[39] Anonymous (2011) China's largest inland lake Poyang Lake turns into grassland in worst drought. Available: http://www.whatsonxiamen.com/news19375.html. Accessed 10 November 2013.

[40] Koh H-S, Ochs CA, Yu K (2009) Hydrologic gradient and vegetation controls on CH4 and CO2 fluxes in a spring-fed forested wetland. Hydrobiologia 630: 271–286.

[41] Wang ZP, DeLaune RD, Patrick WH, Masscheleyn PH (1993) Soil Redox and pH Effects on Methane Production in a Flooded Rice Soil. Soil Science Society of America Journal 57: 382–385.

[42] Swamy YV, Nikhil GN, Venkanna R, Das SN, Chaudhury GR (2012) Emission of methane and nitrous oxide from Vigna mungo and Vigna radiata legumes in India during the dry cropping seasons. Atmosfera 25: 107–120.

[43] Kludze HK, Delaune RD (1995) Gaseous exchange and wetland plant-response to soil redox intensity and capacity. Soil Science Society of America Journal 59: 939–945.

[44] Kolka RK, Thompson JA (2007) Wetland Geomorphology, Soils, and Formative Processes. In: Batzer DP, Sharitz RR, editors. Ecology of Freshwater and Estuarine Wetlands. Berkeley, CA: University of California Press.

[45] Bernal B, Mitsch W (2013) Carbon sequestration in freshwater wetlands in Costa Rica and Botswana. Biogeochemistry 115: 77–93.

[46] Schimel J (2000) Global change: Rice, microbes and methane. Nature 403: 375–377. 10667774 10.1038/35000325

[47] Fazli P, Man HC, Shah UKM, Idris A (2013) Characteristics of Methanogens and Methanotrophs in Rice Fields: A Review. Asia-Pacific Journal of Molecular Biology and Biotechnology 21: 3–17.

[48] Liu DY, Ding WX, Jia ZJ, Cai ZC (2011) Relation between methanogenic archaea and methane production potential in selected natural wetland ecosystems across China. Biogeosciences 8: 329–338.

[49] Li CF, Zhou DN, Kou ZK, Zhi Sheng Z, Jin Ping W, et al (2012) Effects of tillage and nitrogen fertilizers on CH4 and CO2 emissions and soil organic carbon in paddy fields of central China. PLoS ONE 7: e34642. 22574109 10.1371/journal.pone.0034642 PMC3344821

[50] Kammann C, Grünhage L, Jäger HJ, Wachinger G (2001) Methane fluxes from differentially managed grassland study plots: the important role of CH4 oxidation in grassland with a high potential for CH4 production. Environmental Pollution 115: 261–273. 11706799 10.1016/s0269-7491(01)00103-8

[51] Sakin E (2012) Organic carbon organic matter and bulk density relationships in arid-semi arid soils in Southeast Anatolia region. African Journal of Biotechnology 11: 1373–1377.

[52] Chaudhari PR, Ahire DV, Ahire VD, Chkravarty M, Maity S (2013) Soil Bulk Density as related to Soil Texture, Organic Matter Content and available total Nutrients of Coimbatore Soil. International Journal of Scientific and Research Publications 3: 1–8.

[53] Allaire SE, Lafond JA, Cabral AR, Lange SF (2008) Measurement of gas diffusion through soils: Comparison of laboratory methods. Journal of Environmental Monitoring 10: 1326–1336. 18974902 10.1039/b809461f

[54] Hirota M, Tang Y, Hu Q, Hirata S, Kato T, et al (2004) Methane emissions from different vegetation zones in a Qinghai-Tibetan Plateau wetland. Soil Biology and Biochemistry 36: 737–748.

[55] Bhullar GS, Edwards PJ, Olde Venterink H (2013) Variation in the plant-mediated methane transport and its importance for methane emission from intact wetland peat mesocosms. Journal of Plant Ecology 6: 298–304.

[56] Haynes WM, Lide DR, Bruno TJ (2012) CRC Handbook of Chemistry and Physics 2012-2013. Boca Raton, Florida: CRC press.

[57] Huang J, Tong C, Liu Z, Xiao H, Zhang L (2011) Plant-mediated Methane Transport and Emission from a Spartina alterniflora Marsh(in chinese). Chinese Bulletin of Botany 46: 534–543.

[58] Enrica P, Rossano B, Marco B, Pierluigi V (2010) Net primary production and seasonal CO2 and CH4 fluxes in a Trapa natans L. meadow. Journal of Limnology 69: 225–234.

[59] Duan X, Wang X, Zhiyun O (2006) Plant-mediated CH4 emission from a Phragmites-dominated wetland in an arid region, China. Journal of Freshwater Ecology 21: 139–145.

[60] Gauci V, Gowing DJG, Hornibrook ERC, Davis JM, Dise NB (2010) Woody stem methane emission in mature wetland alder trees. Atmospheric Environment 44: 2157–2160.

